# Supersulfides: A Promising Therapeutic Approach for Autoinflammatory Diseases

**DOI:** 10.1111/1348-0421.13205

**Published:** 2025-02-16

**Authors:** Tianli Zhang, Touya Toyomoto, Tomohiro Sawa, Takaaki Akaike, Tetsuro Matsunaga

**Affiliations:** ^1^ Center for Integrated Control, Epidemiology and Molecular Pathophysiology of Infectious Diseases Akita University Akita Japan; ^2^ Department of Microbiology, Graduate School of Medical Sciences Kumamoto University Kumamoto Japan; ^3^ Department of Environmental Medicine and Molecular Toxicology Tohoku University Graduate School of Medicine Sendai Japan

**Keywords:** autoinflammatory diseases, pattern‐recognition receptor, persulfides, polysulfides, reactive sulfur species, supersulfides

## Abstract

Supersulfides are molecular species characterized by catenated sulfur moieties, including low‐molecular‐weight and protein‐bound supersulfides. Emerging evidence suggests that these molecules, abundantly present in diverse organisms, play essential roles far beyond their chemical properties, such as functions in energy metabolism, protein stabilization, and antiviral defense. Recent studies highlight their regulatory effects on pattern‐recognition receptors (PRRs) and associated signaling pathways–such as nucleotide oligomerization domain‐like receptor signaling, toll‐like receptor signaling, and type I interferon receptor signaling–critical for innate immunity and inflammatory responses. Dysregulation of these pathways is implicated in a heterogeneous group of autoinflammatory diseases, including inflammasomopathies, relopathies, and type I interferonopathies, respectively. Notably, both endogenous and synthetic supersulfide donors have recently shown promising inhibitory effects on PRR signaling, offering their potential as targeted therapies for managing autoinflammatory conditions. This review summarizes the fundamental biology of supersulfides and typical autoinflammatory diseases, focusing on their roles in innate immune and inflammatory responses, while exploring their therapeutic potential in these diseases.

**Abbreviations**
AP1: activator protein 1
CAPS: cryopyrin‐associated periodic syndrome
CARS: cysteinyl‐tRNA synthetase
CysSH: cysteine
CysSSH: cysteine persulfide
DAMPs: damage‐associated molecular patterns
FCAS: familial cold autoinflammatory syndrome
FMF: familial mediterranean fever
GSH: glutathione
GSSH: glutathione persulfide
GSSSG: glutathione trisulfide
HA20: haploinsufficiency of A20
HIDS: hyperimmunoglobulin D syndrome
IFN‐I: type I interferon
IFNAR: type I interferon receptor
IKKs: inhibitor kappa B kinases
IL: interleukin
iNOS: inducible nitric oxide synthase
IRAK: IL‐1 receptor‐associated kinases
IRF3: interferon regulatory factor 3
ISGs: interferon‐stimulated genes
IκB*α*
: nuclear factor kappa B‐inhibitory protein
JAK: janus kinase
LMW: low‐molecular‐weight
LPS: lipopolysaccharide
MAVS: mitochondrial antiviral signaling protein
MDA5: melanoma differentiation‐associated gene 5
MKK: mitogen‐activated protein kinase kinase
MVK: mevalonate kinase
MyD88: myeloid differentiation primary response 88
NAC‐S2: N‐acetylcysteine tetrasulfide
NF‐κB: nuclear factor kappa B
NLR: nucleotide oligomerization domain‐like receptor
NLRP: nucleotide oligomerization domain‐like receptor protein
NOD: nucleotide oligomerization domain
PAMPs: pathogen‐associated molecular patterns
PRRs: pattern‐recognition receptors
RIG‐I: retinoic acid‐inducible gene I
RIPK: receptor interacting protein kinase
RLRs: RIG‐I‐like receptors
ROS: reactive oxygen species
STAT: signal transducer and activator of transcription
STING: stimulator of interferon genes
TGS4: thioglucose tetrasulfide
TIR: toll/interleukin‐1 receptor
TIRAP: TIR domain‐containing adapter protein
TLRs: Toll‐like receptors
TNF: tumor necrosis factor
TRAF6: TNF‐associated factor 6
TRIF: TIR domain‐containing adapter‐inducing interferon‐β

## 1 Introduction

**Figure 1:**
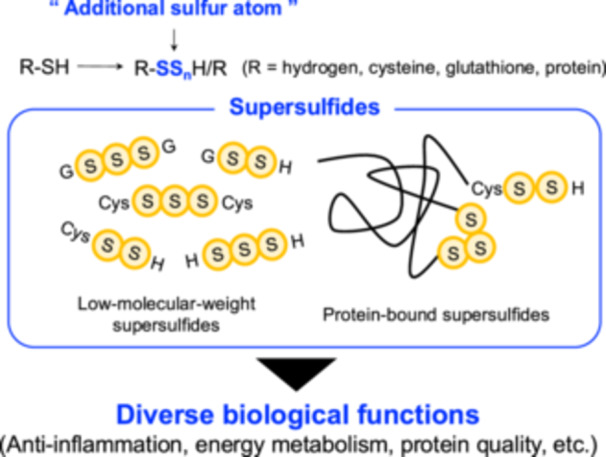
Supersulfides play various roles in biological systems. Supersulfides, a species with catenated sulfur moieties, existing as both LMW and protein‐bound forms. These species play diverse essential functions in biological systems, such as inflammation, energy metabolism, and protein stabilization.

Supersulfides are defined by their catenated sulfur moieties, including hydropersulfides (RSSH), hydropolysulfides (RSS_n_H, *n* > 1), polysulfides (RSS_n_R, *n* > 1), and inorganic persulfides and polysulfides [1]. Quantitative analyses have revealed that these molecules are widespread across prokaryotic and eukaryotic organisms, such as yeast, bacteria, and mammals. Low‐molecular‐weight (LMW) supersulfides derived from cysteine (CysSH)‐containing compounds are of particular interest. These include cysteine persulfide/polysulfide (CysSSH/CysSS_n_H, *n* > 1), glutathione persulfide/polysulfide (GSSH/GSS_n_H, *n* > 1), cysteine trisulfide (Cys‐SSS‐Cys) and glutathione trisulfide (GSSSG). In addition, inorganic supersulfides, such as hydrogen persulfide (H_2_S_2_) and trisulfide (H_2_S_3_), have also been identified in biological contexts. Supersulfides are present in protein‐bound forms as well, where they are linked to thiols of CysSH residues [1]. Beyond their established antioxidant properties, supersulfides are increasingly recognized as critical mediators in diverse physiological and pathological processes. These include regulating protein functions, participating in energy metabolism, modulating immune responses, and preventing ferroptosis [2, 3, 4] (Figure 1). Recent studies have further highlighted their potent anti‐inflammatory properties, particularly in modulating pattern recognition receptors (PRRs) and their associated signaling pathways. Dysregulation of PRR signaling pathways has been strongly implicated in the pathogenesis of autoinflammatory diseases, such as inflammasomopathies, relopathies, and type I interferonopathies, underscoring the importance of targeting these pathways [5, 6, 7]. These findings point to the therapeutic potential of supersulfides in mitigating autoinflammatory conditions associated with such diseases. While the methodology and chemistry of supersulfides have been extensively reviewed elsewhere and fall beyond the scope of this review, readers are encouraged to consult related reviews for a more detailed exploration of these aspects [8, 9, 10]. Here, we focus on the basic biological significance of supersulfides, emphasizing their roles in innate immunity and inflammation. Additionally, we discuss how supersulfides may serve as promising therapeutic candidates.

## 2 Overview of Supersulfides

These supersulfides, irrespective of their forms, can be generated through enzymatic or chemical processes. Initially, cystathionine β‐synthase, cystathionine γ‐lyase, and 3‐mercaptopyruvate sulfurtransferase were believed to be the key enzymes responsible for supersulfide generation [11, 12]. These enzymes catalyze the conversion of CysSH to CysSSH, which can undergo disproportionation to form oxidized species, such as CysSSSCys. Similarly, glutathione (GSH) reacts with CysSSH to produce GSSH, which undergoes analogous transformations. Additionally, GSSH can also be produced through the glutathione reductase‐mediated reduction of oxidized glutathione polysulfides [11]. Moreover, 3‐mercaptopyruvate sulfurtransferase has been shown to generate both LMW and protein‐bound supersulfides via the transsulfuration pathway [12]. However, a study using mice with a triple knockout of these enzymes demonstrated that supersulfides are still produced, indicating the existence of compensatory or alternative mechanisms for supersulfide biosynthesis. Instead, cysteinyl‐tRNA synthetase (CARS) has since been identified as a primary cysteine persulfide synthase, regardless of the forms in which supersulfides exist [13]. Recombinant CARS from various species, including mouse CARS1, human CARS2, and *Escherichia coli* CARS, has been shown to produce CysSSH in the presence of the substrate l‐cysteine [2]. In mammalian cells, two isoforms of CARS exist: cytosolic CARS1 and mitochondrial CARS2. Mass spectrometry‐based analyses revealed that supersulfide levels are significantly reduced in CARS2 knockout cells. Consistently, tissues from heterozygous CARS2 knockout mice exhibited markedly lower levels of LMW supersulfides compared to wild‐type mice [2]. These findings suggest CARS as a principal enzyme for supersulfide synthesis, and evolutionarily conserved from prokaryotes to eukaryotes.

Within mitochondria, supersulfides generated by CARS2 can participate in sulfur‐oxygen hybrid respiration as part of the electron respiratory chain. This process is mainly mediated by sulfide:quinone oxidoreductase, which catalyzes the oxidation of supersulfides [14, 15]. The resulting oxidized supersulfides and their metabolites are further oxidized to sulfite through enzymatic reactions catalyzed by sulfur oxidation enzymes, including persulfide dioxygenase (also known as ETHE1), rhodanese, and sulfite oxidase [16].

**Figure 2:**
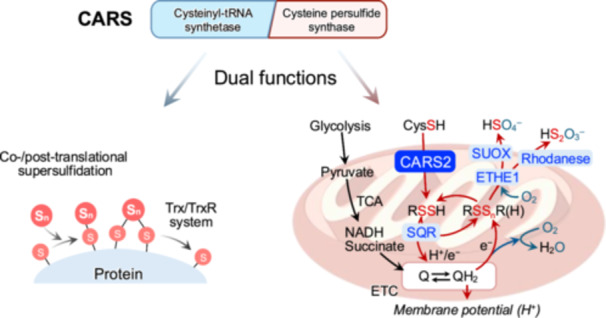
Dual functions of CARS. CARS catalyzes the formation of tRNA‐bound CysSSH adducts, enabling the incorporation of CysSSH into proteins, which reduced by Trx/TrxR system. These dual functions of CARS are integral to cellular sulfur metabolism and redox regulation. Additionally, CARS also catalyzes the CysSSH production through utilizing CysSH as the substrate. This activity contributes to sulfur‐oxygen hybrid respiration within mitochondria. ETHE1, persulfide dioxygenase; NADH, nicotinamide adenine dinucleotide; SQR, sulfide:quinone oxidoreductase; SUOX, sulfite oxidase; TCA, tri‐carboxylic acid cycle; Trx, thioredoxin; TrxR, thioredoxin reductase system.

Notably, through its aminoacyl‐tRNA synthetase activity, CARS can directly incorporate CysSSH into nascent polypeptide chains by conjugating CysSSH with tRNA [2]. This process facilitates the formation of protein‐bound supersulfides during protein translation [2, 17]. Although direct evidence linking supersulfidation to specific protein functions remains limited, the thioredoxin/thioredoxin reductase system has been shown to regenerate protein thiols from protein‐bound supersulfides. This supersulfidation‐depersulfidation switch plays a critical role in both protecting and restoring protein function under conditions of excessive oxidative stress [18, 19] (Figure 2).

## 3 Autoinflammatory Diseases and PRR Signaling

The term “autoinflammation” is often contrasted with “autoimmunity”, which refers to diseases caused by dysfunction in adaptive immune responses that are antigen‐dependent and mediated by autoantibodies or autoreactive T lymphocytes [20]. In contrast, autoinflammatory diseases represent a heterogeneous group of disorders characterized by dysregulation of the innate immune system. These conditions primarily involve sterile inflammation driven by antigen‐independent hyperactivation of immune pathways [21]. Advancements in genomic studies over the past decade have led to a surge in the identification of new autoinflammatory disorders. To date, more than 50 monogenic autoinflammatory diseases are cataloged in the Infever database (https://infevers.umai-montpellier.fr/web/), with new categories continually emerging [22]. However, the identification of variants of unknown significance–mutations not previously linked to disease cohorts–poses challenges in unraveling the complex mechanisms underlying autoinflammatory diseases [23].

From the perspective of the host, inflammatory responses begin with the recognition of specific molecular patterns and are prompted by the production of pro‐inflammatory meditators [24]. For instance, innate immune cells identify pathogen‐associated molecular patterns (PAMPs), such as viral RNA or bacterial lipopolysaccharide (LPS), as well as damage‐associated molecular patterns (DAMPs) released by damaged tissues [25, 26]. These molecules are detected by four types of PRRs, including Toll‐like receptors (TLRs) and C‐type lectin receptors on the cell surface, and nucleotide oligomerization domain (NOD)‐like receptors (NLRs) and retinoic acid‐inducible gene I (RIG‐I)‐like receptors (RLRs) within cytoplasm [27]. These recognitions trigger signal transduction pathways, culminating in the release of pro‐inflammatory mediators and the onset of inflammation [28].

**Figure 3:**
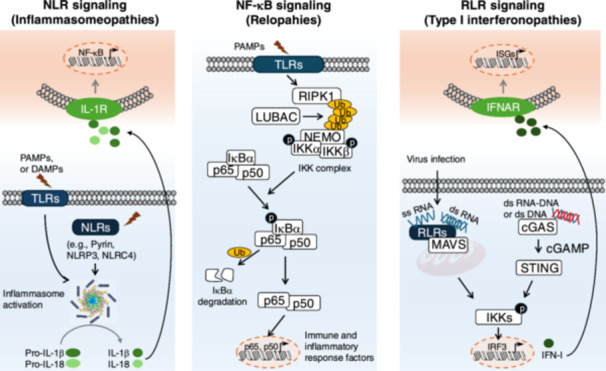
PRR signaling pathways associated with autoinflammatory diseases. Represents three of the main categories: NLR signaling‐mediated inflammasomopathies, NF‐κB signaling‐mediated relopathies, and RIG‐I signaling‐mediated type I interferonopathies. When NLRs recognize their ligands, the inflammasome is formed, which is responsible for the maturation of pro‐inflammatory cytokines, including IL‐1β and IL‐18. Since then, both cytokines are released into the extracellular space and recognized by IL‐1 receptors of other cells, triggering the NF‐kB signaling pathway; The NF‐κB signaling can be activated by diverse stimuli. This activation elicits the production of inflammatory mediators; The recognition of double‐stranded DNA or RNA‐DNA hybrids by cGAS activates the production of cGAMP, which binds to STING and subsequently triggers IKK signaling. On the other hand, the binding of single‐stranded or double‐stranded RNA to RLRs (e.g., RIG‐I) promote its association with MAVS within the mitochondria, leading to IRF3 phosphorylation and the production of IFN‐I. The released IFN‐I can further bind to IFNAR, leading to the upregulation of ISGs. cGAMP, 2′‐3′‐cyclic AMP‐GMP; cGAS, cyclic GMP‐AMP synthase; LUBAC, linear ubiquitin chain assembly complex; NEMO, NF‐κB essential modulator; Ub, ubiquitination.

Current dogma holds that the pathogenesis of most autoinflammatory diseases is closely associated with dysregulation of PRR‐mediated signaling pathways, leading to excessive production of inflammatory mediators. Examples include the overproduction of tumor necrosis factor (TNF)‐α via TLR signaling, type I interferon (IFN‐I) via RLR signaling, and interleukin (IL)‐1β via NLR signaling [29]. Such dysregulation is often caused by gene mutations, leading to either a loss of function in anti‐inflammatory genes or a gain of function in pro‐inflammatory genes. These mutations result in unchecked inflammatory responses, giving rise to various disease subtypes, including relopathies, type I interferonopathies and inflammasomopathies [30] (Figure 3).

## 4 Typical Examples of Autoinflammatory Diseases

Initial reports of patients with distinct phenotypes laid the foundation for understanding autoinflammatory diseases. However, the identification of additional patients sharing common genotypes has revealed a far broader spectrum of these conditions than initially recognized (Table 1). Below, we discuss representative examples of monogenetic autoinflammatory diseases.

**Table 1:** typical autoinflammatory diseases.

Subtypes	Name	Gene	Protein	Phenotypes	Pathogenesis
Inflammasomopathies	FMF	*MEFV (NM_000243.2)*	Pyrin	Fever (12–72 h), serositis, non‐erosive acute arthritis of large joints, erysipelas‐like lower extremity rash	Gain‐of‐function mutations causing poor affinity to regulatory proteins (protein kinase N 1/2, 14‐3‐3)
	PAAND	*MEFV (S242R)*	Pyrin	Fever, neutrophilic dermatosis, acne, pyoderma gangrenosum, cutaneous abscesses	Loss of pyrin inhibition by 14‐3‐3 protein
	MKD (HIDS)	*MVK (NM_000431.4)*	Mevalonate kinase	Early‐onset (< 1 year), fever (3–7 days), GI symptoms, arthromyalgia or arthritis, maculo‐papular or urticarial rash, aphthous stomatitis, hepatosplenomegaly, cervical adenopathy	Prenylation of proteins, necessary for Rho‐A activation and PI3K‐mediated inhibition of pyrin inflammasome
	PAPA	*PSTPIP1 (NM_003978.5)*	CD2‐binding protein 1	Pyoderma gangrenosum, arthritis, acne	Gain‐of‐function mutations of CD2‐BP1, which enhance pyrin inflammasome activation
	PFIT	*WDR1 (NM_017491.5)*	WD40 repeat protein 1	Fever (up to 7 days), mucosal ulcerations, thrombocytopenia, infections	Hypomorphic mutation, actin accumulation, pyrin inflammasome activation dysregulation
	CAPS	*NLRP3 (NM_004895.4)*	Cryopyrin/NLRP3	FCAS: cold‐triggered episodes of fever, urticaria, conjunctivitis; MWS: cold‐induced urticaria, sensorineural hearing loss; CINCA: neonatal onset, urticaria, chronic aseptic meningitis, deforming arthropathy, facial dysmorphia	Gain‐of‐function mutations of NLRP3 inflammasome
	FCAS2	*NLRP12 (NM_144687.3)*	Monarch 1/NLRP12	Fever, cold‐induced urticaria, arthralgia	Constitutive NF‐κB activation
	FCAS4	*NLRC4 (NM_001199138.2)*	NLRC4	Neonatal onset, cold‐induced urticaria, arthralgia, fever	Gain‐of‐function mutations, hyperactivation of NLRC4 inflammasome
	AIFEC	*NLRC4 (NM_001199138.2)*	NLRC4	Early‐onset enterocolitis, recurrent MAS	NLRC4 inflammasome hyperactivation
Relopathies	TRAPS	*TNFRSF1A (NM_001065.4)*	TNF receptor superfamily member 1A	Fever (> 7–14 days), periorbital edema, conjunctivitis, pseudo‐cellulitis rash, abdominal pain, migrating myalgia, arthralgia, chest pain, lymphadenopathy	Altered intracellular TNFR trafficking
	BS	*NOD2 (NM_001370466.1)*	NOD2/CARD15	< 5 years of age, rash, granulomatous uveitis, symmetrical polyarthritis	Gain‐of‐function mutations
	ORAS	*FAM105B (NM_138348.6)*	OTULIN	Onset < 3 months, fever, diarrhea, arthritis, lipodystrophy, panniculitis, growth restriction	Loss‐of‐function mutations defective deubiquitylation, constitutive hyperactivation of NF‐κB
	HA20	*TNFAIP3 (NM_001270508.2)*	A20	Fever, oral, gastrointestinaland genital ulcerations, arthritis, uveitis (Behcet's disease)	Loss‐of‐function mutations, defective deubiquitylation, constitutive hyperactivation of NF‐κB
	LUBAC deficiency	*HOIL1 (NM_031229.4)* *HOIP (NM_017999.5)*	HOIL1 HOIP	Fever, immunodeficiency, hepatosplenomegaly, amylopectin‐like deposits in muscles	Defective deubiquitylation, constitutive hyperactivation of NF‐κB
Type I interferonopathies	IFIH1	*MDA5 (NM_022168.4)*	Melanoma differentiation‐associated protein 5	SLE, IgA deficiency, mild lower limb	Mutations of IFN‐I Cytosolic sensor for dsRNA
	TREX1	*TREX1 (NM_130384.3)*	Three prime repair exonuclease 1	FCL	Mutations of IFN‐I degradation of intracellular ds‐ss DNA
	SAMHD1	*SAMHD1 (NM_080628.3)*	SAMHD1	FCL	Mutations of IFN‐I cytoplasmic ssRNA/DNA sensor
	SAVI	*STING (NM_198282.4)*	Stimulator of interferon gene	Skin vasculopathy, bilateral interstitial lung disease	Gain‐of‐function mutations of IFN‐I pathway
	PRAAS	*PSMA3/PSMB8, PSMB4/PSMB9, PSMB4/PSMB8, PSMB9 (GRCh38:6:32,854,191‐32,859,850), PSMB10 (GRCh38:16:67,934,505‐67,936,849), PSMB7 (GRCh38:9:124,353,464‐124,415,441), PSMA3 (GRCh38:14:58,244,842‐58,272,003), POMP (GRCh38:13:28,659,129‐28,678,958), PSMG2 (GRCh38:18:12,658,737‐12,725,739)*	Proteasome complex subunit and proteosome chaperone	CANDLE syndrome: chronic neutrophilic dermatosis panniculitis with lipodystrophy, elevated temperature	Loss‐of‐function mutations in proteasome components, causing IFN‐I pathway upregulation
	ISG15 deficiency	*ISG15 (NM_005101.4)*	ISG15	Neurological involvement, mycobacterial susceptibility	Abnormal IFN‐I signaling
	USP18 deficiency	*UDP18 (NM_019076.4)*	UDP18	Neurological involvement, hepatomegaly, thrombocytopenia	Altered inhibition of IFNAR signaling
	SMS	*IFIH1 DDX58 (NM_014314.4)*	IFIH1, DExD/H‐Box, helicase 58	Dental and skeletal dysplasia, aortic calcification, glaucoma and psoriasis	Mutations of a cytosolic receptor for dsRNA

Abbreviations: AIFEC, autoinflammations and infantile enterocolitis; BS, Blau syndrome; IFIH1, interferon induced with helicase C domain 1; LUBAC, linear ubiquitin chain assembly complex; MAS, macrophage activation syndrome; MKD, mevalonate kinase deficiency; MWS, Muckle‐Wells syndrome; ORAS, OTULIN‐related autoinflammatory syndrome; PAAND, pyrin‐associated auto‐inflammation with neutrophilic dermatosis; PAPA, pyogenic arthritis, pyoderma gangrenosum and acne; PFIT, periodic fever immunodeficiency and thrombocytopenia; PRAAS, proteasome associated autoinflammatory syndromes; SAMHD1, sterile alpha motif domain and histidine‐aspartate domain containing deoxynucleotide triphosphate triphosphohydrolase 1; SAVI, STING‐associated vasculopathy with onset in infancy; SMS, singleton merten syndrome; TNFAIP3, TNF‐induced protein 3; TRAPS, TNF receptor‐associated periodic syndrome; TREX1, three prime repair exonuclease 1; USP, ubiquitin‐specific protease.

### 4.1 Inflammasomopathies

NOD‐like receptor protein (NLRP) subfamily, represents the largest and most extensively studied group of NLRs, which play a crucial role not only in sensing PAMPs/DAMPs, but also in detecting disturbances in cellular homeostasis, such as redox imbalance and cellular stress [31]. Most NLRPs are involved in immune and inflammatory responses through the assembly of a multiprotein platform known as the inflammasome [32]. Despite minor variations depending on the specific NLRP involved, inflammasomes are involved in host defense by promoting the maturation of pro‐inflammatory cytokines, including IL‐1β and IL‐18 [33] (Figure 3). Genetic mutations that lead to dysregulated NLRs or inflammasome‐mediated cytokine overproduction are collectively referred to as inflammasomopathies.

The first recognized autoinflammatory disease is an inflammasomopathy, with familial mediterranean fever (FMF) reported in 1945 and characterized by phenotypes including episodic fever, serositis, elevated inflammatory markers, and amyloidosis [34]. FMF is caused by autosomal recessive gain‐of‐function mutations in the *MEFV* gene, which encodes the protein pyrin, a component of inflammasomes [35]. Mutations such as V726A, E148Q, M694V, M680I and M694I account for approximately 70% of FMF cases, with M680I and M694I associated with more phenotypes [35]. These mutations generally impair the binding of 14‐3‐3 to protein kinase N 1/2, effector kinases of RhoA, thereby reducing pyrin phosphorylation. This disruption activates the pyrin inflammasome, leading to excessive IL‐1β secretion [36].

Another pyrin inflammasome‐related disorder is mevalonate kinase (MVK) deficiency, also known as hyperimmunoglobulin D syndrome (HIDS). This rare autosomal recessive disease results from loss‐of‐function mutations in the *MVK* gene on chromosome 12 [37]. More than 140 pathogenic *MVK*‐associated variants have been identified, with V377I being the most common [38]. Most HIDS patients are compound heterozygous, carrying two distinct mutations in each allele. Dysfunction of MVK enhances pyrin inflammasome activity through reducing RhoA geranylgeranylation. Clinically, the disease manifests as recurrent fever, abdominal pain, and skin and gastrointestinal symptoms, often triggered by infections or vaccinations during infancy [39, 40].

The NLRP3 inflammasomes is among the most extensively studied inflammasomes [41]. Cryopyrin‐associated periodic syndrome (CAPS) arises from autosomal dominant or de novo gain‐of‐function mutations in the middle domain of *NLRP3* gene. These mutations impair the binding of regulatory molecules such as cyclic AMP and caspase recruitment domain‐containing protein 8, resulting in hyperactivation of NLRP3 inflammasome [42]. In addition, CAPS can also occur due to myeloid lineage‐specific somatic mutations in the *NLRP3* gene or mosaicism [43, 44]. CAPS includes three clinical subtypes of increasing severity: familial cold autoinflammatory syndrome (FCAS), Muckle‐Wells syndrome, and neonatal‐onset multisystem inflammatory disease. Symptoms range from fever, urticaria, and arthritis to chronic aseptic meningitis, sensorineural hearing loss, and severe developmental abnormalities [42].

Patients with mutations in NLRC4, another member of the NLR family, present during infancy with enterocolitis and recurrent macrophage activation syndrome. This disease is characterized by high‐grade fevers, hepatosplenomegaly, lymphadenopathy, sepsis‐like manifestations, and elevated serum ferritin, which can progress to organ damage and death if untreated [45]. Gain‐of‐function mutations causing autoinflammation with infantile enterocolitis localize to the NOD region of NLRC4, leading to spontaneous oligomerization and constitutive secretion of IL‐1β and IL‐18 [46]. Consequently, IL‐18 levels are significantly elevated in the serum of patients, driving disease symptoms [47].

### 4.2 Relopathies

Relopathies are autoinflammatory conditions associated with dysregulated nuclear factor kappa B (NF‐кB) signaling. Normally, NF‐κB activation is triggered by PAMPs binding to TLRs. This initiates a signaling cascade involving the LUBAC, which generates linear polyubiquitin chains and ligates ubiquitin to receptor interacting protein kinase 1 (RIPK1) and NEMO upon receptor complex formation. These polyubiquitination events lead to the phosphorylation of inhibitor kappa B kinases (IKKs), including IKKα and IKKβ, which located upstream of NF‐κB‐inhibitory protein (IκBα). Subsequently, IKKs phosphorylate IκBα, resulting in its degradation. As a result, the NF‐κB subunits p50 and p65 are released and translocate into the nucleus, where they drive the production of inflammatory mediators [48] (Figure 3).

Biallelic loss‐of‐function mutations in *RIPK1* gene lead to RIPK1 deficiency, whereas monoallelic mutations cause cleavage‐resistant RIPK1‐induced autoinflammatory syndrome. Variants such as p.Leu321Arg and p.Asp324Gly impair RIPK1 cleavage by caspase 8, perpetuating NF‐κB‐mediated signaling [49]. Blau Syndrome is a rare autosomal dominant condition caused by gain‐of‐function mutations in *NOD2* gene. NOD2 recognizes bacterial muramyl‐dipeptide and self‐oligomerizes, subsequently interacting with RIPK2 and triggering NF‐κB activation.

Another relopathy, OTULIN‐related autoinflammatory syndrome, involves mutations in the catalytic deubiquitinase domain of OTULIN. These mutations result in dysregulated NF‐κB activity and elevated circulating levels of TNF‐α and IL‐6. Patient monocytes exhibit exaggerated cytokine responses to LPS stimulation, including increased secretion of IL‐1β and TNF‐α, as compared to healthy controls [50]. Furthermore, haploinsufficiency of A20 (HA20) results from autosomal dominant missense mutations in *TNF‐induced protein 3* gene. These mutations reduce its production and impair deubiquitylation, leading to constitutive NF‐κB activation. Notably, HA20 predominantly affects individuals in Japan and typically manifests during childhood with severe systemic inflammation [51].

### 4.3 Type I Interferonopathies

Type I interferonopathies are characterized by chronic activation of the RLR‐IFN‐I axis. Upon virus invasion, RIG‐I and melanoma differentiation‐associated gene 5 (MDA5), typical members of RLRs, are activated and interacted with mitochondrial antiviral signaling protein (MAVS) [52]. This interaction triggers downstream signaling and production of IFN‐I, including IFN‐α and IFN‐β. Furthermore, secreted IFN‐I binds to type I interferon receptor (interferon‐α/β receptor) (IFNAR), activating the Janus kinase–signal transducer and activator of transcription (JAK–STAT) signaling pathway and inducing the expression of IFN‐stimulated genes (ISGs) [53]. Additionally, aberrant nucleic acid sensing, accumulation of endogenous nucleic acids, or dysregulated IFNAR signaling also contribute to this disorder [54] (Figure 3).

Aicardi‐Goutières syndrome manifests as progressive encephalopathy, recurrent fevers, and livedo reticularis, often leading to severe disabilities [55]. This disorder involves mutations in over 40 genes, most commonly including loss‐of‐function variants in *ADAR, RNU7‐1, LSL11, TREX1, SAMHD1, RNASEH2A, RNASEH2B, and RNASEH2C* genes, and gain‐of‐function mutations in *IFIH1* gene, which effect on either accumulation of nucleic acid or activity of MDA5 [55]. Similarly, mutations in *USP18* gene, that limits IFNAR signaling, leads to an Aicardi‐Goutières syndrome‐like syndrome, known as pseudo‐toxoplasmosis, rubella, cytomegalovirus and herpes syndrome [56]. Stimulator of interferon genes (STING)‐associated vasculopathy with onset in infancy arises from gain‐of‐function mutations in the *TMEM173* gene, which encodes the STING protein. This hyperactivation leads to excessive production of IFN‐I, IL‐6, and TNF‐α, causing systemic inflammation, vasculopathy and interstitial lung disease [57].

## 5 Therapeutic Potential of Supersulfides in Autoinflammatory Diseases

**Figure 4:**
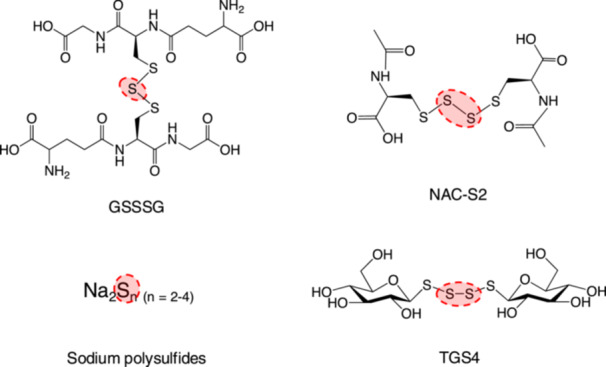
Chemical structures of supersulfide donors. The chemical structures of supersulfide donors introduced in this review.

Clinically, nonsteroidal anti‐inflammatory drugs are often employed as symptomatic treatment for pain and inflammation in autoinflammatory diseases, either alone or in combination with baseline therapy. However, their limited efficacy and associated risks have been observed in most majority of patients [30]. Recently, supersulfide donors, including GSSSG, N‐acetylcysteine tetrasulfide (NAC‐S2), thioglucose tetrasulfide (TGS4), and inorganic polysulfide sodium salts (e.g., disodium di‐, tri‐, and tetra‐sulfide; Na_2_S_n_), have been shown to elevate cellular supersulfide levels and regulate PRR‐mediated signaling pathways [7, 10] (Figure 4). This section introduces these supersulfide donors with therapeutic potential for treating autoinflammatory conditions.

### 5.1 Inhibition of NLRP3 Inflammasome‐Mediated Cytokine Production

A large‐scale genome‐wide association study demonstrated that CARS2 deletion significantly increased the production of inflammatory cytokines in macrophages exposed to LPS [58]. Furthermore, studies using CARS2 heterozygous knockout mice infected with various viruses revealed exacerbated disease severity compared to wild‐type mice, as evidenced by lung histopathology and elevated cytokine production, including TNF‐α, IL‐1β and IL‐6 [3]. Since CARS2 is the major enzyme for supersulfide synthesis, these findings suggest that supersulfides act as suppressor of inflammation. Supporting this, macrophages lacking the cystine transporter xCT showed significantly lower levels of CysSSH after LPS stimulation compared to wild‐type macrophages [59]. Under the same conditions, upregulation of pro‐inflammatory genes (e.g., *IL‐1b*) in macrophages was observed, further indicating the inhibitory effect of supersulfides on NLRP3 inflammasome activation.

**Figure 5:**
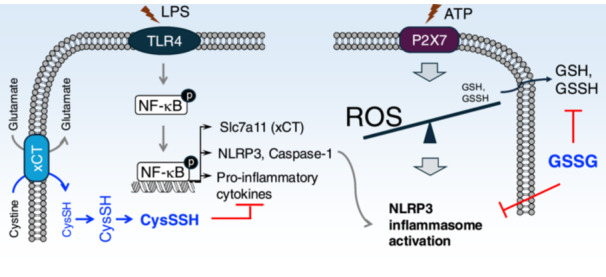
Effects of supersulfides on NLRP3 inflammasome activation. In activated macrophages, *slc7a11*, which encodes the xCT, is significantly upregulated. This leads to enhance cystine uptake, promoting the production of CysSSH and establishing a negative feedback loop to limit excessive inflammatory mediators. On the other hand, ATP exposure stimulates rapid GSH and GSSH efflux via the purinergic P2X7 receptor, leading to an imbalance with subsequent activation of the NLRP3 inflammasome. GSSG strongly inhibits the NLRP3 inflammasome activation through restoring cellular GSSH.

Although the precise molecular mechanisms remain to be fully elucidated, reactive oxygen species (ROS) are widely regarded as essential for the activation of the NLRP3 inflammasome [60]. Indeed, Zhang et al. demonstrated that ATP exposure triggers rapid GSH and GSSH excreted from LPS‐primed macrophages, resulting in ROS accumulation and redox imbalance, which collectively lead to NLRP3 inflammasome activation. Simultaneously, the extracellular addition of oxidized GSH (GSSG) suppressed the efflux of GSSH, strongly inhibiting NLRP3 inflammasome‐mediated IL‐1β release [6]. Furthermore, a recent study emphasized that supplementation with CysSSH protects macrophages from DAMPs‐induced pyroptosis by modulating NLRP3 persulfidation, providing further valuable insights into the regulatory role of supersulfides in inflammasome activation [61] (Figure 5).

### 5.2 Suppression of TNF‐α Production Through Inhibiting NF‐κB Phosphorylation

**Figure 6:**
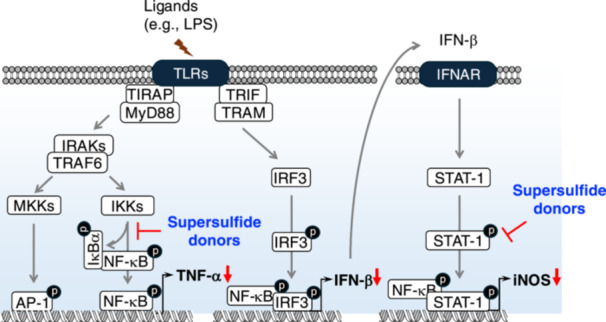
Inhibitory effect of supersulfides on NF‐κB signaling. Upon ligand exposure, TLRs trigger the production of pro‐inflammatory mediators, including TNF‐α, IFN‐β, and iNOS, through the phosphorylation of NF‐κB signaling. NAC‐S2 potently inhibits the NF‐κB phosphorylation, thereby suppressing the downstream inflammatory responses. AP‐1, activator protein 1; TIRAP, TIR domain‐containing adapter protein; TRAM, TIR domain‐containing adapter‐inducing interferon‐β–related adapter molecule.

TLR4 signaling can be categorized into myeloid differentiation primary response 88 (MyD88)‐dependent and Toll/interleukin (IL)‐1 receptor (TIR) domain‐containing adapter‐inducing interferon‐β (TRIF)‐dependent pathways [62]. MyD88‐dependent signaling directly via TIR domain‐containing adapter protein (TIRAP) leads to the assembly of IL‐1 receptor‐associated kinases (IRAK) and tumor necrosis factor (TNF)‐associated factor 6 (TRAF6), culminating in the phosphorylation of IKKs [63]. As described above, IKKs phosphorylate IκBα, making it for degradation and thereby releasing NF‐κB. Consequently, NF‐κB translocate to the nucleus to generate pro‐inflammatory cytokines, such as TNF‐α [64]. Concurrently, activation of the mitogen‐activated protein kinase kinase (MKK) cascades, leading to activator protein 1 (AP1) activation, another critical transcription factor in inflammation [65]. In the TRIF‐dependent pathway, TRIF‐related adapter molecule facilitates the recruitment of TRIF, triggering the production of IFN‐I through the phosphorylation of IFN regulatory factor 3 (IRF3). IFN‐I further signals in both autocrine and paracrine manners by binding to IFNAR. This binding activates the STAT1 phosphorylation, which eventually triggers inducible nitric oxide synthase (iNOS) expression [66] (Figure 6).

Cell‐based analyses showed that NAC‐S2, a synthetic supersulfide donor, significantly suppressed LPS‐induced NF‐κB phosphorylation in macrophages. However, NAC‐S2 did not inhibit the adjacent MKK‐mediated signaling pathway, which suggests that supersulfides specifically target NF‐κB signaling [5]. This study also found that NAC‐S2 completely inhibited downstream inflammatory factors, including TNF‐α. Similar as NAC‐S2, TGS4, another synthetic supersulfide donor, also significantly suppressed TNF‐α production under the same conditions [7]. Additionally, the inorganic supersulfide donor Na_2_S_4_ limited NF‐κB activation through modulating IKKβ [67]. In addition to TLR4, NAC‐S2 also strongly inhibited other TLR‐mediated NF‐κB signaling, as evidenced by suppression of TNF‐α production from macrophages stimulated with ligands for TLR2 and TLR3 (Figure 6). Beyond these synthetic supersulfide donors, GSSSG, another endogenous supersulfie donor, was shown to suppress LPS‐induced inflammatory profiling in both mouse and human epithelial cells [68]. Importantly, administration of LPS to mice decreased the survival rate to 20% due to lethal septic shock. However, treatment of mice with NAC‐S2 increased survival rate to 90% and ameliorated inflammatory responses, such as reduced circulating TNF‐α [5].

### 5.3 Blockage of IFN‐I Signaling Pathway by Supersuflides

As above‐mentioned, persistent activation of TLR4 leads to IFN‐β production, which subsequently induces iNOS expression through the STAT1‐mediated signaling pathway during the late phase of inflammation. This pathway was further examined in LPS‐treated RAW264.7 cells, where IFN‐β production, accompanied by STAT1 phosphorylation, was detected. Notably, either iNOS expression or IFN‐I production was abolished when RAW264.7 cells were treated with LPS in the presence of NAC‐S2 or TGS4 [5, 7] (Figure 6).

**Figure 7:**
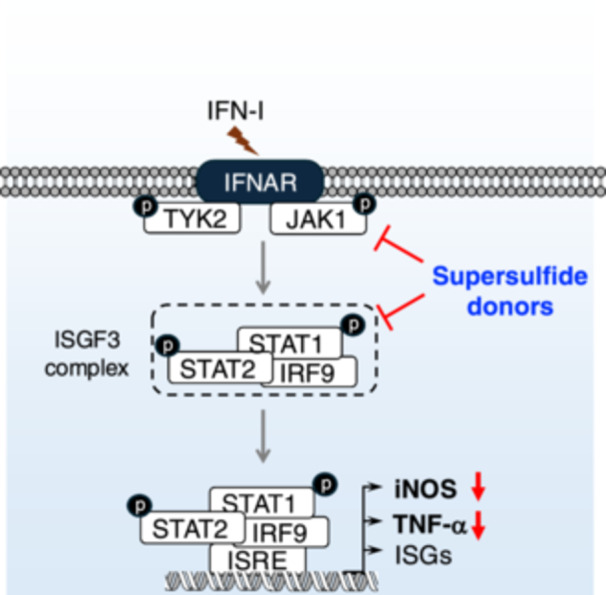
Suppression of IFN‐I signaling by supersulfides. Binding of IFN‐I to their receptors phosphorylates JAK1 and TYK2, resulting in the release of phosphorylated STAT1/2 heterodimer. The dimer binds to the transcription factor IRF9 to form the ISGF3 complex, that translocate to nucleus and binds to the promoter region of ISRE to activate the transcription of ISGs. Elevation of intracellular supersulfides by NAC‐S2 blocks this signaling by inhibiting JAK1/STAT1 phosphorylation, resulting in the reduction of ISGs expression. ISGF3, IFN‐stimulated gene factor 3; ISRE, IFN‐stimulated response element; TYK2, tyrosine kinase 2.

During viral infection, recognizing of IFN‐I by IFNAR activates the intracellular JAK–STAT pathway, particularly STAT1 and STAT2 [66, 69]. STAT phosphorylation forms an IFN regulatory factor 9‐bound complex, which amplifies ISGs expression [70]. Interestingly, supersulfide donors abolished IFN‐I signaling in macrophages stimulated not only with IFN‐α but also with IFN‐β. Specifically, both NAC‐S2 and TGS4 strongly suppressed STAT1 phosphorylation, likely due to potent inhibition of upstream JAK1 phosphorylation (Figure 7). It is also noteworthy that NAC‐S2 also exhibited inhibitory effects on IFN‐γ‐induced signaling, highlighting the broader inhibitory effects of supersulfides on IFN signaling.

Taken together, this evidence strongly supports that supersulfides primarily exert their therapeutic effects on autoinflammatory conditions by targeting these keys signaling pathways.

## 6 Concluding Remarks

In this review, we introduced supersulfides, which play a crucial role in life‐sustaining phenomena across organisms. As described, the multifaceted anti‐inflammatory effects of supersulfides underscore their protective functions against autoinflammatory diseases and their potential for clinical application. In this emerging field, a deeper understanding of the mechanisms of action, molecular targets, pharmacokinetics, and potential side effects in disease models is essential. Also, to enhance the efficacy of therapeutic agents, developing drug delivery systems that enable supersulfide donors to specifically target affected areas is critically important.

## Author Contributions

Writing–original draft preparation: Tianli Zhang and Touya Toyomoto. Writing–review and editing: Tianli Zhang. Figures: Tianli Zhang and Touya Toyomoto. Supervision: Tetsuro Matsunaga, Tomohiro Sawa, and Takaaki Akaike. Funding acquisition: Tetsuro Matsunaga, Tomohiro Sawa, and Takaaki Akaike. All authors have read and agreed to the published version of the manuscript.

## Disclosure

The authors declare no conflicts of interest.

## Data Availability

The data that support the findings of this study are available from the corresponding author upon reasonable request.
